# Heterogeneous Expression of the Core Circadian Clock Proteins among Neuronal Cell Types in Mouse Retina

**DOI:** 10.1371/journal.pone.0050602

**Published:** 2012-11-26

**Authors:** Xiaoqin Liu, Zhijing Zhang, Christophe P. Ribelayga

**Affiliations:** 1 Department of Ophthalmology and Visual Science, The University of Texas Health Science Center at Houston, Medical School, Houston, Texas, United States of America; 2 The University of Texas Health Science Center at Houston, Graduate School of Biomedical Sciences, Houston, Texas, United States of America; Morehouse School of Medicine, United States of America

## Abstract

Circadian rhythms in metabolism, physiology, and behavior originate from cell-autonomous circadian clocks located in many organs and structures throughout the body and that share a common molecular mechanism based on the *clock* genes and their protein products. In the mammalian neural retina, despite evidence supporting the presence of several circadian clocks regulating many facets of retinal physiology and function, the exact cellular location and genetic signature of the retinal clock cells remain largely unknown. Here we examined the expression of the core circadian clock proteins CLOCK, BMAL1, NPAS2, PERIOD 1(PER1), PERIOD 2 (PER2), and CRYPTOCHROME2 (CRY2) in identified neurons of the mouse retina during daily and circadian cycles. We found concurrent clock protein expression in most retinal neurons, including cone photoreceptors, dopaminergic amacrine cells, and melanopsin-expressing intrinsically photosensitive ganglion cells. Remarkably, diurnal and circadian rhythms of expression of all clock proteins were observed in the cones whereas only CRY2 expression was found to be rhythmic in the dopaminergic amacrine cells. Only a low level of expression of the clock proteins was detected in the rods at any time of the daily or circadian cycle. Our observations provide evidence that cones and not rods are cell-autonomous circadian clocks and reveal an important disparity in the expression of the core clock components among neuronal cell types. We propose that the overall temporal architecture of the mammalian retina does not result from the synchronous activity of pervasive identical clocks but rather reflects the cellular and regional heterogeneity in clock function within retinal tissue.

## Introduction

Circadian clocks orchestrate metabolism, physiology, and behavior with respect to the 24-h rotations of the Earth and the associated variations in the external world. These internal timekeeping mechanics provide living beings with the adaptive advantage of anticipating and preparing for the daily geophysical fluctuations of their environment [1]. The core machinery of circadian clocks is a well-conserved cellular mechanism based on a set of genes-the *clock* genes- and their protein products-the *clock* proteins- interlocked in transcriptional-translational feedback loops that self-regenerate with a period close to 24-h [1]. In mammals, fundamental elements of the clock mechanism have been identified. These include the transcription activators CLOCK, NPAS2, and BMAL1 and their inhibitors PERIOD (PER) and CRYPTOCHROME (CRY) [2].

Many, if not all, aspects of the physiology and function of the vertebrate retina vary on a daily basis. These include photoreceptor disk shedding, gene expression, the synthesis and release of neurohormones and neurotransmitters (such as melatonin and dopamine), neuronal light responses, and components of the electroretinogram [3], [4], [5], [6]. Importantly, most of these rhythms persist in constant conditions (*i.e.* constant darkness) with a period of approximately 24 h, reflecting their control by endogenous circadian clocks [3], [4], [5], [6]. The formal demonstration that the vertebrate retina contains a circadian clock came from the classic *in vitro* work on retinal and photoreceptor melatonin by Cahill and Besharse [7], [8]. Those works on *Xenopus* were then followed by essentially similar papers on mouse retina [9], [10], [11]. In mammals however, notwithstanding intense research, our knowledge of the origin of retinal circadian rhythms remains incomplete. In particular, despite widespread clock gene expression in the retinal tissue [4], [5] and functional evidence supporting the presence of a clock in the photoreceptor layer [9], [10], [11] and in the inner retina [12], [13], [14], it is still unknown whether the clock components are expressed in most or in specific retinal cells. To date, the only retinal cell type in which concurrent expression of the core clock components has been consistently observed is the dopaminergic amacrine cell [12], [15], [16], [17]. In addition, it is still largely unknown whether rhythms of clock gene transcript expression translate into rhythms of clock protein accumulation in retinal cells.

In an attempt to identify the circadian clock neurons in mouse retina, we used a semi-quantitative immunocytochemical approach to investigate the expression of six key circadian clock proteins in a number of retinal neurons labeled with specific markers. Our data indicate that the core clock elements are expressed in most neurons in the mouse retina and reveal a large degree of homogeneity within a same cell type and of heterogeneity between cell types not only in the amount but also in the rhythmic occurrence of clock protein expression. This important disparity in clock protein expression among cell types suggests that circadian rhythms in the retina are built upon distinct subpopulations of neuronal cellular clocks. Our observations raise the possibility that the strong heterogeneity we observed in the retina, and that others have observed in the suprachiasmatic nucleus of the hypothalamus (SCN) [18], is a general feature of circadian clock organization in mammalian tissues.

## Materials and Methods

### Animals and Lighting Conditions

This study was carried out in strict accordance with the recommendations in the Guide for the Care and Use of Laboratory Animals of the National Institute of Health. The protocol was approved by the Animal Welfare Committee of the University of Texas Health Science Center at Houston (Protocol Number: HSC-AWC-09-095, renewed 12–043). Most of the experiments were conducted on adult (1–6 months) C57Bl/6J mice of either sex supplied by the Jackson Laboratory (stock 000664; Bar Harbor, MA). In addition, some of the experiments were conducted on *Opn4^Cre^*; *R26^Z/G^* mice that express enhanced green fluorescence protein (eGFP) in the melanopsin-expressing intrinsically photosensitive ganglion cells ipRGCs. These mice were created and given to us by Dr. Samer Hattar (The Johns Hopkins University, [19]). We also used retinal tissue from *Clock^−/−^* mice [20], *Npas2^−/−^* [21], *Per1^−/−^*, and *Per2^−/−^* mice (described in [22]) (gifts from Dr. David R. Weaver, University of Massachusetts), and rodless and coneless mice (gifts from Dr. Benjamin E. Reese, University of California at Santa Barbara). The rodless mice were created by Dr. Anand Swaroop (National Eye Institute) and are deficient for the transcription factor neural retina-specific leucine zipper protein (NRL), which is critical for rod differenciation and homeostasis. *Nrl^−/−^* mice have a retina with predominantly S-opsin-containing cones that exhibit molecular and functional characteristics of wild-type cones. Because *Nrl^−/−^* retinas start to degenerate at about one month of age, we used retinas from 3 to 4 week old mice [23]. The coneless mice were created by Dr. Jeremy Nathans (The Johns Hopkins University) by expressing an attenuated diphtheria toxin under the control of flanking sequences from the human L-cone opsin gene. Fully mature coneless retinas lack most of the cones (∼95%) but otherwise appear structurally quite normal [24]. We used 3 to 4 week old coneless mice.

Mice were housed in groups of 5 in polycarbonate cages and entrained for at least 2 weeks to a 12-h light/12-h dark (LD) cycle with lights on at 7∶00 a.m. prior to an experiment. Circadian (DD) conditions were created by keeping the mice in the dark for up to 36 h, with dark adaptation starting at the end of the light phase (7.00 p.m.). We refer to the light phase between 7∶00 a.m. and 7∶00 p.m. as the *day*, also defined as the period between Zeitgeber Time (ZT) 0 and ZT12, and the dark phase between 7∶00 p.m. and 7∶00 a.m. as the *night* or the period between ZT12 and ZT24. Accordingly, under circadian conditions, we refer to the *subjective day* as the period between Circadian Time (CT) 0 and CT12 that is, between 12 and 24 h after the beginning of dark adaptation, and the *subjective night* as the period between CT12 and CT24, that is 24 and 36 h after the beginning of dark adaptation. Manipulation of the animals, tissue collection, and fixation were performed under room lights during the day and under infrared light during the night or under circadian conditions with the help of infrared goggles (D-321G-A; Night Optics USA, Hungtington Beach, CA).

### Tissue Collection

Mice were anesthetized with a mixture of ketamine and xylazine (100/10 mg/kg, I.M.), decapitated, and their eyes rapidly collected and placed in phosphate buffered saline (PBS) pH 7.3. Two cuts were made in the cornea under a dissecting microscope and under infrared illumination with dual-unit Prowler Night Vision scopes (Meyers Electro Optics, Redmond, WA). The eyes were then fixed in 4% paraformaldehyde in 0.1 M PBS at 4°C for 1 h. After a quick rinse in PBS, the fixed eyeballs were placed overnight in 30% sucrose in PBS at 4°C. Cryoprotected eyes were embedded in optimum cutting temperature formulation, flash frozen, and cut in 12 µm sections in a cryostat. Sections were placed on glass slides and dried for 30 min at 40°C in a hybridization oven and kept at −80°C until use.

### SDS-PAGE and Western Blotting

C57Bl/6J mouse neural retinas were dissected between ZT09 and ZT12 under room lights. Ten freshly dissected retinas were immersed in 5 volumes of lysis buffer A (20 mM HEPES, pH 7.4, 2 mM ethylenediaminetetraacetic acid [EDTA], 2 mM DL-dithiothreitol [DTT], 0.2 mM phenylmethylsulfonyl fluoride (PMSF), 1x Protease Inhibitor Cocktail (P8340; Sigma-Aldrich Corp., St. Louis, MO)], and immediately homogenized on ice with an ultrasonic dismembrator (model 150E; Fisher Scientific, Pittsburg, PA). The homogenate was mixed with 5 volumes of Buffer B (20 mM HEPES, pH 7.4, 0.2 M sodium chloride [NaCl], 2% Triton X-100), and incubated at 4°C with agitation for 30 minutes and centrifuged at 20,000 rpm for 30 minutes at 4 °C. The supernatant was collected and mixed with 2x Laemmli sample buffer (161–0737; Bio-Rad Laboratories, Hercules, CA). Protein samples were separated on Mini-PROTEAN TGX Gels (any kD, 456–9034S; Bio-Rad Laboratories) and transferred electrophoretically to nitrocellulose membranes (Micron Separations, Westborough, MA). The membranes were incubated in blocking buffer (5% milk in Tris-buffered saline [TBS]) for 2 h at room temperature. All subsequent incubations were performed in blocking buffer. The following primary antibodies were used: rabbit anti-CLOCK (1∶1000; AB2203; Millipore, Billerica, MA), rabbit anti-BMAL1 (1∶4000; R37), rabbit anti-NPAS2 (1∶6000; R35 final), rabbit anti-PER1 (1∶500; AB5424P; Millipore), rabbit anti-PER2 (1∶500; AB5428P; Millipore), or rabbit anti-CRY2 (1∶500; CRY21-A; Alpha Diagnostics, San Antonio, TX) (see Table 1 for full description of the antibodies). The blots were probed with a DyLight488-conjugated donkey anti-rabbit secondary antibody (1∶2000; 711-485-152; Jackson ImmunoResearch Laboratories, West Grove, PA), and the signals detected by a variable mode imager (Typhoon 9400; Amersham Biosciences; Piscataway, NJ). Alternatively, a peroxidase-conjugated secondary antibody (1∶5000; 711-035-152; Jackson ImmunoResearch Laboratories) was used with a chemiluminescence detection system (cat# 34080; SuperSignal West Pico Chemiluminescent substrate, Pierce Biotechnology Inc., Rockford, IL).

**Table 1:** List of the antibodies used in this study.

Antibody	Source/dilution	References/additional controls
CLOCK (Rb α-mouse CLOCK)*	Chemicon-Millipore, AB2203, 1∶1000	[20], [22], [25]/no staining in *Clock^−/−^* retina; similar pattern of labeling with R40 or GP89x (1∶16,000; gifts from Dr. Weaver)
BMAL1 (Rb α-mouse BMAL1)*	gift from Dr. Weaver, R37, 1∶5,000	[22], [25]/similar pattern of labeling with GP85x (1∶16,000; gift from Dr. Weaver)
NPAS2 (Rb α-mouse NPAS2)*	gift from Dr. Weaver, R35, 1∶5,000	[20]/very weak staining in *NPas2^−/−^* retina; similar pattern of labeling with GP84 (1∶8,000; gift from Dr. Weaver)
PER1 (Rb α-human PERIOD1) *	Chemicon-Millipore, AB5424p, 1∶500	[25]/no staining following the pre-adsorption of the synthetic peptide AG330 (Chemicon); similar pattern of labeling with 1117 or R43 (1∶16,000; gift from Dr. Weaver) or EX0005 (1∶500)
PER1 (GP α-mouse PERIOD1)	KeraFAST Inc., EX0005, 1∶500	[20], [25]/no staining in *Per1^−/−^* retina; similar pattern of labeling with 1117 or R43 (1∶16,000; gift from Dr. Weaver) or AB5424p (1∶500)
PER2 (Rb α-mouse PERIOD2)*	Chemicon-Millipore, AB5428p, 1∶200	[25]/weak staining in *Per2^−/−^* retina; no staining following the pre-adsorption of the synthetic peptide AG332 (Chemicon); similar pattern of labeling with GP87 (1∶8,000; gift from Dr. Weaver) or EX0006 (1∶500)
PER2 (GP α-mouse PERIOD2)	KeraFAST Inc., EX0006, 1∶500	[22], [25]/no staining in *Per2^−/−^* retina; similar pattern of labeling with GP87 (1∶8,000; gift from Dr. Weaver) or AB5428p (1∶200)
CRY2 (Rb α-mouse CRYPTOCHROME2)*	Alpha Diagnostic Intl., CRY21-A, 1∶100	[17], [25]/no staining following the pre-adsorption of the synthetic peptide CRY21-P (Alpha Diagnostic Intl.)
Antibody	Source**/**dilution	Cell type marker
cARR (Gt α-cone arrestin)	Santa Cruz Biotechnology, sc-54355, 1∶500	cone photoreceptors**
Blue opsin (Rb α-blue opsin)	Chemicon-Millipore, AB5407, 1∶200	short-wavelength (blue) cone photoreceptors
CALD28 (Ms α-calbindin D-28K)	Sigma-Aldrich Co. LLC, C9848, 1∶5,000	horizontal cells
CHX10 (Shp α-CHX10)	Exalpha Biologicals, Inc., X1180P, 1∶300	bipolar cells
TH (Shp α- tyrosine hydroxylase)	Chemicon-Millipore, AB1542, 1∶5,000	dopaminergic amacrine cells
ChAT (Gt α-choline acetyl transferase)	Chemicon-Millipore, AB144P, 1∶200	cholinergic (Starburst) amacrine cells
Pax6 (Ms α-paired box protein 6)	Chemicon-Millipore, MAB5552, 1∶100	amacrine cells+ganglion cells
Brn3b (Gt α-brain-specific homeobox/POUdomain protein 3b)	Santa Cruz Biotechnology, sc-6026, 1∶200	ganglion cells
Melanopsin (Rb α-melanopsin)	Adv Target Sys., AB-N38, 1∶5,000	ipRGCs
GFP (Ck α-green fluorescent protein)	Aves, GFP-1020, 1∶1,000	eGFP+ cells (ipRGCs)

* : antibodies against clock proteins that we used for the semi-quantitative analysis of clock protein expression in mouse retina.

** : we determined that the cARR antibody labeled all cones in the mouse retina, included the blue cones, in double labeling experiments with the blue opsin antibody (Chemicon-Millipore, AB5407).

### Immunohistochemistry

A comprehensive list of all the antibodies used in this study is given in Table 1. We were able to detect and study CLOCK, BMAL1, NPAS2, PER1, PER2, and CRY2 expression in the mouse retina. We did not find an antibody to CRY1 that gave acceptable staining, nor did we test antibodies to proteins not considered *bona fide* components of the mammalian clock (e.g. DEC1). For immunofluorescent labeling, retinal sections were washed for 5 min in PBS and treated with 10% methanol (in PBS) for 5 min, blocked for 2 h in 5% donkey serum/0.3% Triton X-100 (in PBS), and incubated overnight at room temperature with a primary antibody against one of the clock proteins. Following incubation with the primary antibody, sections were rinsed in PBS (10×5 min) and reacted with a secondary antibody conjugated to Dylight488 or Dylight 549 (1∶600; Jackson ImmunoResearch Laboratories) for 2 h at room temperature in the dark. Finally, sections were covered with a DAPI fluorescent mounting medium and sealed with nail polish. In double labeling experiments, sections were first reacted with a primary antibody against a cell marker (see Table 1 for a complete list). After reaction with the secondary antibody, they were washed in PBS (10×5 min) and reacted with a primary antibody against a clock component. The order of this sequence proved to be the best to preserve clock protein immunogenicity.

Although the specificity of each of the antibodies to clock components used in this study has been previously characterized by others (Table 1), we performed a series of controls to confirm the previous results. Control experiments included (1) omission of one or both primary antibodies, resulting in labeling by the remaining primary antibody or nonspecific background staining (data not shown); (2) Western blot analysis of protein extracts from mouse retinas. The results were in agreement with previous studies [25] and the molecular weight of the clock protein (data not shown); (3) testing the antibody in an animal in which the clock gene had been mutated (*Clock^−/−^*, *Npas2^−/−^*, *Per1^−/−^*, *Per2^−/−^*) or following the pre-adsorption of the synthetic peptide against which the antibody was raised (see Table 1 for details); and (4) comparing the staining pattern obtained with two antibodies against different portions of the same molecule (see Table 1 for details). The specificity of the immunocytochemical markers has been extensively described by others in the field of retinal circuitry (see for example [26]). In our hands, each of these antibodies only stained cells with the classic morphology and distribution described by others, and therefore no further characterization of their specificity was pursued.

### Image Acquisition and Data Analysis

Images were digitally captured using a confocal microscope (Zeiss LSM510; Carl Zeiss Microscopy, LLC, Thornwood, NY) equipped with a laser diode (405 nm) and argon (488 nm), helium/neon (546 nm) and helium/neon (633 nm) lasers. Because the thickness of the mouse retina and variation of the densities of neurons between central and peripheral retina [27], images were collected at randomly selected locations in the region from 20 to 80% of the distance from the optic nerve head to the periphery. However, ganglion cell (GC) density in the mouse retina decreases from a peak near the optic nerve head towards the periphery. Specifically, in 20–80% of retinal eccentricity the decrease in GC density is approximately 1.5 in B6 mice (Dr. Steven W. Wang, personal communication). To obtain a reliable measure of the section mean fluorescence intensity, and because variation in the number of GCs with eccentricity (and thus per section) could have introduced some noise in the measurements, we selected sections with a similar number of cells in the GC layer (GCL). However, to quantify the clock protein immunosignal in cells of the GCL, we measured the fluorescence intensity within the boundary of each cell (visualized by DAPI staining). With this method, the mean average intensity was independent of the number of cells per section. We did not observe any obvious differences in immunosignal intensity between different areas (at different eccentricities) of the retina, in agreement with a previous study [15]. Three confocal slices at 0.4 µm intervals were stacked to generate a 1,200 dpi/12 bit tiff image. Rhythmic intensity analysis of each clock protein required us to perform a preliminary screening of the sections to determine the maximum intensity and the brightest pixel intensity so that the intensity of the majority of pixels fell in the linear range of the intensity curve. The settings, including final magnification of the image, were then kept unchanged when images were acquired from other sections. Five images were collected for each eyecup, with images distributed among three to four sections. Each eyecup was collected from a different animal, and at least five animals were sampled for each experimental condition.

### Statistical Analysis

Quantification of clock protein-related fluorescence was performed using the SimplePCI software (Version 5.3.0.1102, Compix Inc. Imaging Systems, Cranberry Township, PA). Mean fluorescence intensity of clock protein expression within a retinal section was determined within a squared area whose upper and lower boundaries were the outer and inner limiting membranes, respectively. To count the number of cells per retinal layer that expressed the clock proteins, cells were visualized with DAPI staining of their nucleus, and the immunofluorescent signal was quantified. Clock protein expression in the cell was considered significant when the fluorescence value was higher than 150% of the background value. Clock protein expression within an identified retinal neuron was determined by measuring the mean fluorescence intensity within the boundaries of the cell soma labeled for a specific marker for all the somas within a section. All the cells from a given type within a section were indiscriminately subjected to quantification, whether they appeared to express clock proteins or not. Mean fluorescence in rods was determined within a large area (∼50×20 µm) selected in the outer nuclear layer (ONL) that did not include the cone somas. Background fluorescence was measured by placing a 50×20 µm rectangle on the inner plexiform layer (IPL) and was subtracted to each individual value. However, we found that the immunofluorescence level in rods and cones was often lower than that in the IPL, even though it was found rhythmic in cones. As we established constitutively low expression for any clock protein in rods (rod signal <5% of section mean fluorescence) and because rods and cones have similar shape and volume, we used the mean fluorescence level measured in the rods as the value of background that was subtracted from the cone value.

Statistics was performed with Prism 5 for Windows (Version 5.01, GraphPad Software, Inc.). Specific immunoflurorescence measurements from individual cells from the same type from 3 to 4 sections from the same animal were tested for normality using the Kolmogorov-Smirnov test and averaged. Data from 5 to 6 animals were averaged for each time point and also tested for normality. We then determined whether the expression of a clock component varied with the time of day using a one-way analysis of variance (ANOVA). Clock component expression was considered rhythmic only when *P*<0.05. When rhythmicity was established, to further characterize the rhythm, individual values were fitted to the COSINOR equation:
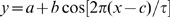
where *y* is the *n*th data point (specific immunofluorescence level), × the time of the *n*th data point (h), *a* the mean (mesor; specific immunofluorescence level), *b* the amplitude (equal to one-half of the sinusoid; specific immunofluorescence level), *c* the acrophase (h), and *τ* the endogenous period (h). For simplification, *τ* was set to 24.0 h. The regression coefficients (*a*, *b*, and *c*) were computed and are given in the text with their respective standard error estimates. *Post hoc* comparison of the regression coefficients between LD and DD cycles was performed using the Student *t*-test. For all analyses performed in the present study, *P<*0.05 was considered significant.

## Results

### Distribution and Temporal Expression Profiles of the Core Mammalian Circadian Clock Proteins in the Mouse Retina

**Figure 1:**
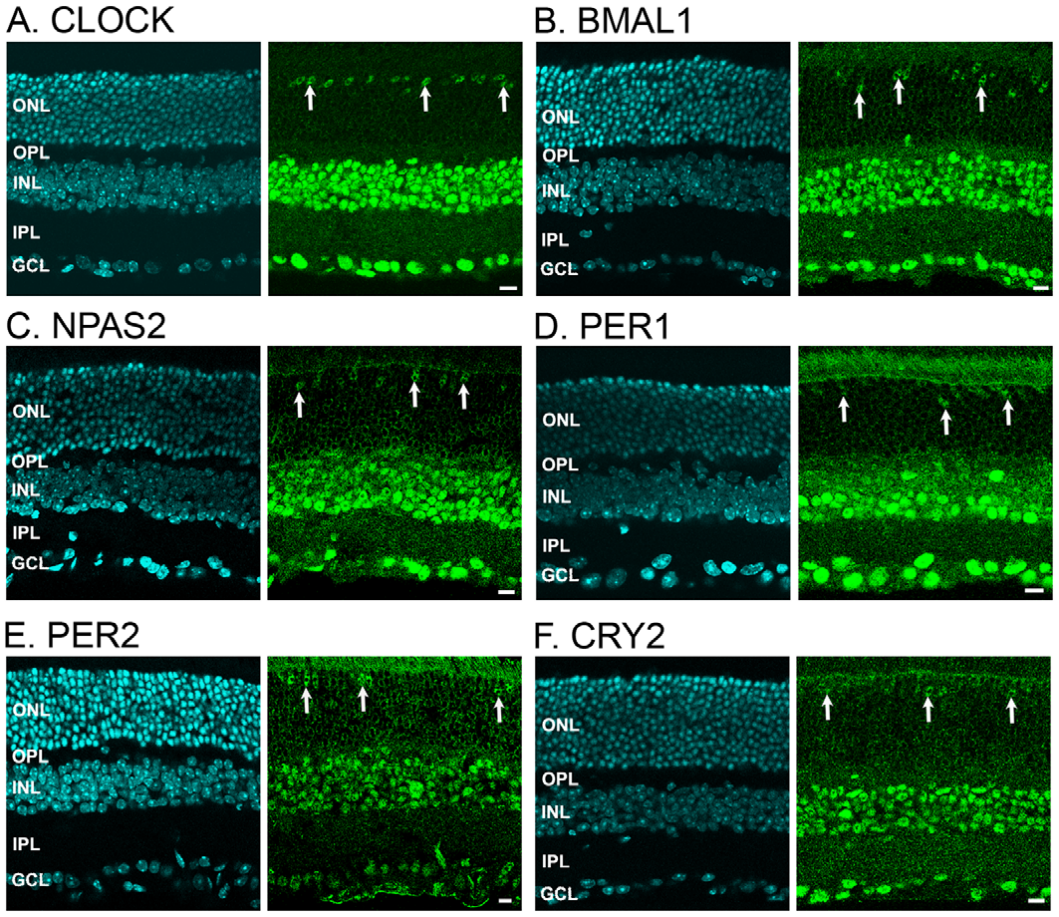
Distribution of six core mammalian circadian clock proteins in the mouse retina. Typical examples of vertical sections of C57Bl/6 mouse retinas collected in the middle of the day during a LD cycle (ZT02-06) are illustrated for CLOCK (***A***), BMAL1 (***B***), NPAS2 (***C***), PER1 (***D***), PER2 (***E***), and CRY2 (***F***). Expression of each clock protein is clearly prominent in the inner nuclear layer (INL) and most cells in the ganglion cell layer (GCL). However, expression is also detected in few cells in the outer nuclear layer (ONL) (vertical arrows). These cells were later identified as cones. OPL: outer plexiform layer; IPL: inner plexiform layer. Optical sections 3×0.4 µm. Bar is 10 µm.

We examined the expression of the core mammalian circadian clock proteins CLOCK, BMAL1, NPAS2, PER1, PER2, and CRY2 in adult C57Bl/6J mouse retinas collected in early morning (ZT02-04) during a LD cycle. Immunolabeling was observed for each clock protein, primarily in the inner retina (Fig. 1). Hence, clock protein immunolabeling was detected in the perikarya of many cells located in the inner nuclear layer (INL) and the ganglion cell layer (GCL). In the outer nuclear layer (ONL), which contains the somatas of rod and cone photoreceptors, immunolabeling was overall lower compared to the inner retina, except within a few cells whose somatas were located in the outermost part of the ONL (Fig. 1). These cells represented ∼3–4% of all the cells in the ONL and were later formally identified as cone photoreceptors (see below). A gradient in fluorescence intensity was clearly observed from the proximal (GCL) to the distal (ONL) areas of the retina for most clock proteins. Regional variations in fluorescence intensity may originate from a variety of reasons, including differences in cell size or cytoplasm volume and may not reflect differences in clock protein expression and/or function. For these reasons we did not pursue the analysis of relative fluorescence intensity between retinal regions or cell types. Rather, we focused on the temporal changes in fluorescence intensity within identified neuronal cell types (see below). The proportion of cells that showed clock protein-related immunofluorescence above background at ZT02-04 was calculated for each nuclear layer and is shown in Table 2.

**Table 2:** Proportion of cells among the retinal nuclear layers that express the core circadian clock components.

nuclear layer	Clock protein					
	CLOCK	BMAL1	NPAS2	PER1	PER2	CRY2
ONL	3.95±0.13%	3.59±0.26%	3.14±0.19%	3.17±0.16%	2.99±0.24%	4.25±0.17%
	(125/3162)	(139/3869)	(123/3914)	(105/3315)	(110/3675)	(123/2895)
INL	91.22±1.74%	86.60±4.94%	88.60±2.76%	62.11±4.97%	81.23±1.98%	81.09±2.09%
	(1029/1128)	(1221/1410)	(1143/1290)	(654/1053)	(870/1071)	(669/825)
GCL	90.74±5.31%	85.63±3.39%	91.46±2.26%	83.33±3.06%	64.62±6.67%	72.00±4.03%
	(147/162)	(149/174)	(225/246)	(165/198)	(126/195)	(108/150)

Retinas were collected around ZT02 during a LD cycle. Cells were identified by DAPI staining of their nucleus. Significant clock protein expression was defined as fluorescent level higher than 150% background. The number of positive cells/total number of cells counted is indicated between parentheses. Data shown are averaged values from 3–5 animals (3 pictures from 3 different sections per animal) ± SEM.

Previous investigations using the green fluorescent protein (GFP) as a reporter for *Per1* expression in the mouse retina described much of the GFP-PER1 labeling in less than 10% of the INL and GCL cells [15]. Because the antibody we used against PER1 was generated against the C-terminus portion of human PER1 (AB5424p; Chemicon-Millipore) and yielded more extensive immunolabeling among retinal cells than in [15] (Table 2), in addition to a series of tests of specificity (see Table 1), we also tested a different antibody directed against the N-terminus part of mouse PER1 (PER1-1-GP; KeraFast; 1∶200). The pattern of immunolabeling of PER1-1-GP was very similar to that of AB5424p (data not shown). A recent study also reported widespread expression of PER1 in the mouse retina [28]. Our observations and others indicate that PER1 expression is consistently broader in mouse retina when assessed by immuncytochemistry than GFP-PER1 expression.

**Figure 2:**
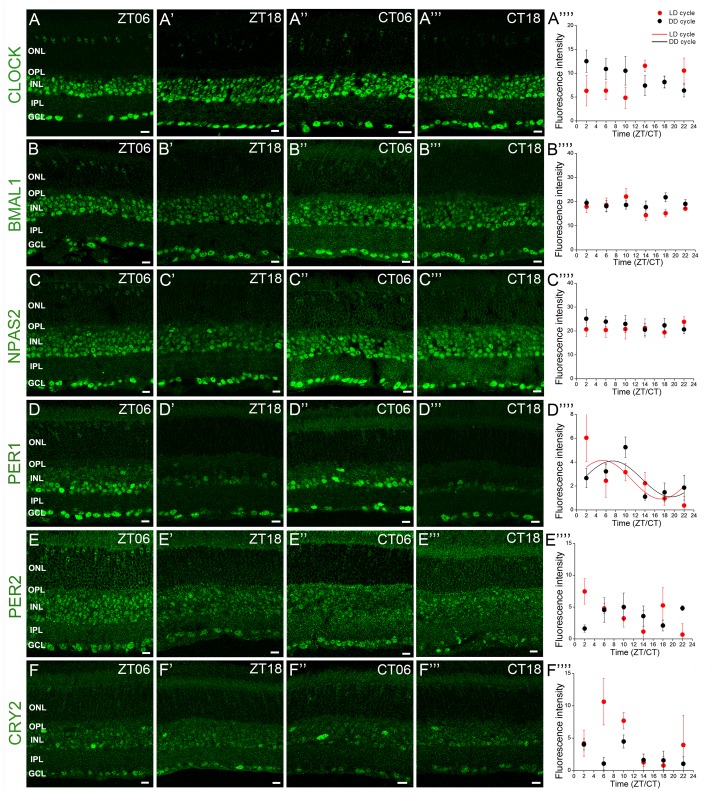
Expression of the six core mammalian circadian clock proteins CLOCK, BMAL1, NPAS2, PER1, PER2 and CRY2 in mouse retina during LD and DD cycles. Typical examples of vertical sections of C57Bl/6 mouse retinas collected in the middle of the day (ZT06; *A–F*) or subjective day (CT06; *A”–F”*) or middle of the night (ZT18; *A’–F’*) or subjective night (CT18; *A”’–F”’*) are illustrated for CLOCK (*A–A”’*), BMAL1 (*B–B”’*), NPAS2 (*C–C”’*), PER1 (*D–D”’*), PER2 (*E–E”’*), and CRY2 (*F–F”’*). Optical sections 3×0.4 µm. Bar is 10 µm. Temporal expression profiles of CLOCK, BMAL1, NPAS2, PER1, PER2, and CRY2 in mouse retinal sections under LD and DD conditions (*A””–F””*). Mouse retinas were collected every 4 h during a LD cycle or a DD cycle, and clock protein expression was quantified by measuring the mean fluorescence per section within a squared area whose upper and lower boundaries were the outer and inner limiting membranes, respectively (see Materials and Methods for details). COSINOR regression was performed only when variations in clock protein expression with time of day were detected (one-way ANOVA; *P*<0.05). Note that only PER1 expression is significantly rhythmic under either LD or DD conditions (*D””*). Each data point represents the mean fluorescence/section +/− SEM of 5/6 animals (5 pictures from 3–4 sections/animal). ONL: outer nuclear layer; OPL: outer plexiform layer; INL: inner nuclear layer; IPL: inner plexiform layer; GCL: ganglion cell layer.

To determine the temporal expression profile of the core clock proteins, retinas were harvested every 4 h during a LD and a DD cycle. Examples of clock protein immunolabeling at different time points of the cycles are shown in Figure 2. The mean fluorescence intensity per retinal section was quantified in a squared area whose upper and lower boundaries were the outer and inner limiting membranes, respectively (Fig. 2; see Material and Methods section for details). The expression of CLOCK, BMAL1, NPAS2, PER2 and CRY2 did not significantly vary during an LD or a DD cycle (Fig. 2). In contrast, PER1 expression was rhythmic under both LD and DD conditions, with a peak of expression around ZT05 and CT07, respectively (Fig. 2*D*).

All together these findings indicate that the six key components of the mammalian circadian clock can be detected in every retinal layer, and the overall expression of most of them (CLOCK, CRY2, BMAL1, NPAS2, PER2) is arrhythmic under both LD and DD conditions, whereas only PER1 is significantly rhythmic under both conditions. These data globally agree with previous studies that reported low amplitude, if any, clock gene expression in the rodent retina [11], [12], [13], [14], [15], [28], [29], [30], [31], [32], [33], [34], [35], [36], [37], [38]. However, there is little information about the exact identity of the clock protein-expressing cells, and it is still unclear whether the overall profile of expression mirrors that within individual retinal cells. After identifying the neuronal cell types that expressed core clock components, we next followed clock protein expression within several cell types during a LD and a DD cycle to test whether the overall temporal profile of expression reflected the expression behavior in individual cells and/or cell types.

### Expression of the Circadian Clock Core Components in Identified Retinal Neurons

**Figure 3:**
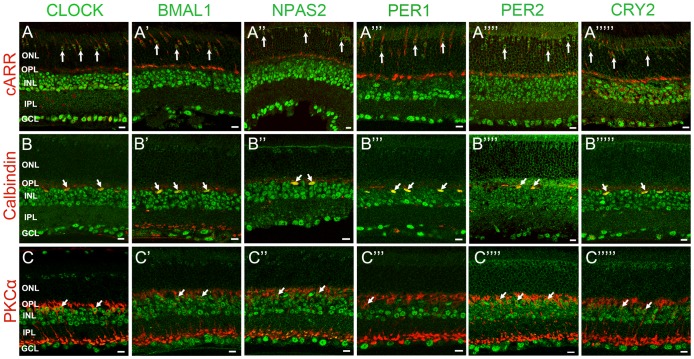
Mammalian core circadian clock protein expression in cones, horizontal cells and rod bipolar cells of the mouse retina. Typical examples of vertical sections of mouse retinas collected between ZT02 and ZT06 and double labeled for one of the following clock proteins: CLOCK (***A*** *–* ***C***), BMAL1 (***A*** *’–* ***C*** *’*), NPAS2 (***A*** *”–* ***C*** *”*), PER1 (***A*** *’”–* ***C*** *’”*), PER2 (***A*** *””–* ***C*** *””*), and CRY2 (***A*** *’””–* ***C*** *’””*) and one of the following protein markers: cARR (cones; ***A***), CALD28 (horizontal cells; ***B***), and PKCα (rod bipolar cells; ***C***). (see Table 1 for details about the antibodies). Note that in the outer nuclear layer (ONL) clock protein expression was evident in cones but was weak or absent in rods. Some double-labeled retinal neurons are shown (arrows). OPL: outer plexiform layer; INL: inner nuclear layer; IPL: inner plexiform layer. Optical sections 3×0.4 µm. Bar is 10 µm.

**Figure 4:**
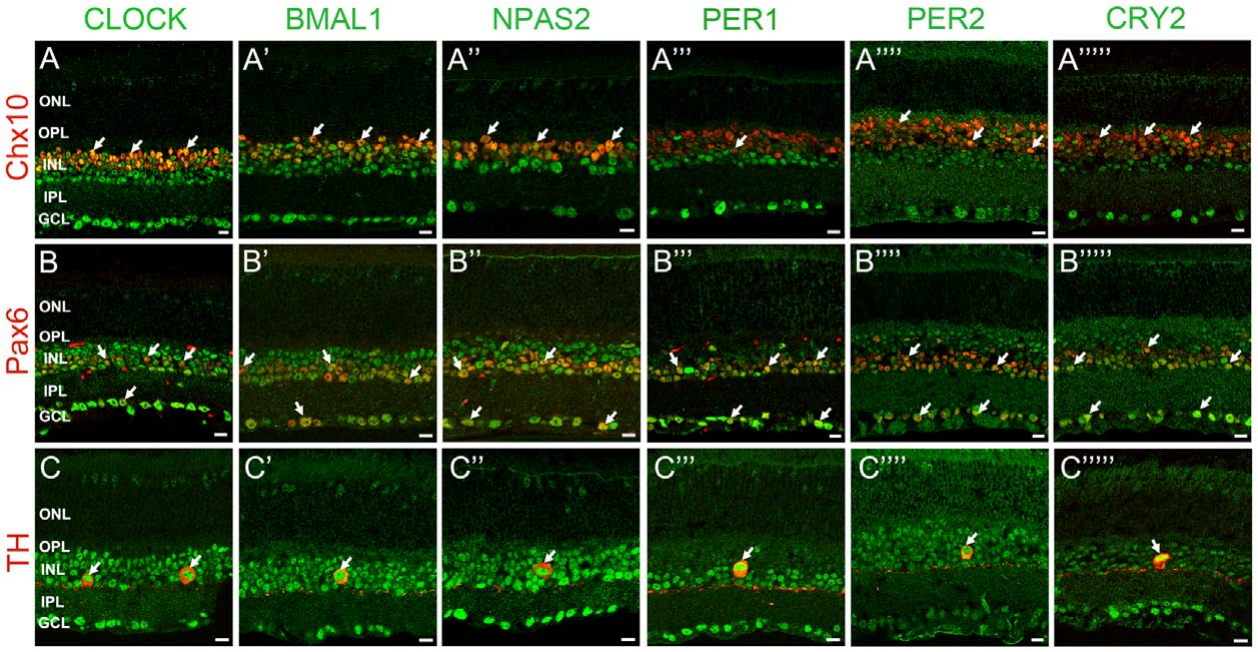
Mammalian core circadian clock protein expression in bipolar, amacrine and ganglion cells of the mouse retina. Typical examples of vertical sections of mouse retinas collected between ZT02 and ZT06 and double labeled for one of the following clock proteins: CLOCK (***A–C***), BMAL1 (***A’–C’***), NPAS2 (***A”–C”***), PER1 (***A’”–C’”***), PER2 (***A””–C””***), and CRY2 (***A’””–C’””***) and one of the following protein markers: Chx10 (bipolar cells; ***A***), Pax6 (most amacrine cells and ganglion cells; ***B***), and TH (dopaminergic amacrine cells; ***C***) (see Table 1 for details about the antibodies). The analysis was restricted to type-1 catecholamine amacrine cells that express high levels of TH. Some double-labeled retinal neurons are shown (arrows). Abbreviations and bar as in Fig. 3.

**Figure 5:**
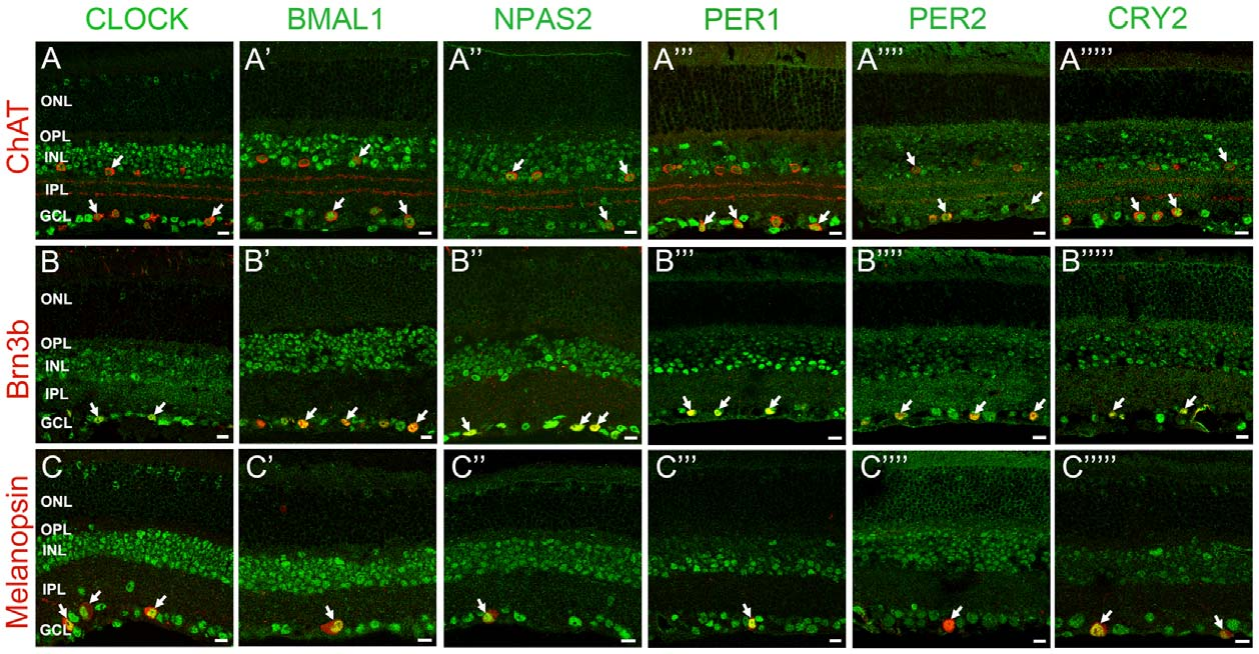
Mammalian core circadian clock protein expression in amacrine and ganglion cells of the mouse retina. Typical examples of vertical sections of mouse retinas collected between ZT02 and ZT06 and double labeled for one of the following clock proteins: CLOCK (***A–C***), BMAL1 (***A’–C’***), NPAS2 (***A”–C”***), PER1 (***A’”–C’”***), PER2 (***A””–C””***), and CRY2 (***A’””–C’””***) and one of the following protein markers: ChAT (starburst amacrine cells; ***A***), Brn3b (most ganglion cells; ***B***), and eGFP (ipRGCs; ***C***) (see Table 1 for details about the antibodies). The concurrent expression of CLOCK, BMAL1, NPAS2, PER1, PER2, and CRY2 was found in all identified neurons. Note also that all the clock proteins are expressed in the ON starburst amacrine cells whose cell body is located in the ganglion cell layer (GCL), indicating that both GCs and displaced amacrine cells in the GCL express the core components of the mammalian clock. Clock protein expression in ipRGCs was confirmed with the AB-N38 antibody (data not illustrated), although this antibody only labeled M1 and M2 ipRGC subtypes. Some double-labeled retinal neurons are shown (arrows). Abbreviations and bar as in Fig. 3.

We examined the distribution of clock protein expression in specific neurons of the mouse retina by double-label immunocytochemistry using antibodies against specific cell markers (see Table 1 for a complete list). In some experiments, we used retinas from the *Opn4^Cre^*; *R26^Z/G^* mouse line that express eGFP in all five subtypes of ipRGCs [19]. IpRGCs express the photopigment melonopsin and represent a third class of photoreceptor in the mammalian retina. These cells play a key role in non-image forming vision. In particular, they convey irradiance information to the SCN and provide photo-entrainment of the SCN clock and control the pupillary light reflex [39], [40]. We investigated clock protein immunolabeling in retinas collected around ZT02-06 in cones (cARR positive cells in the ONL, these include short-wavelength (blue) cones), horizontal cells (CALD28 positive), bipolar cells (Chx10 positive), rod bipolar cells (PKCα positive), amacrine cells (Pax6 positive cells in the INL), dopaminergic amacrine cells (TH positive), starburst amacrine cells (ChAT positive), ganglion cells (Brn3b positive and Pax6 positive cells in the RGC layer), and ipRGCs (melanopsin or eGFP positive). Clock protein expression in rods was determined by measuring the mean intensity of the immunofluorescent signal in an area of the ONL that did not show cARR-immunoreactivity (see Materials and Methods section for details). The expression of each clock protein was significant in most cell types with the notable exception of rods, in which labeling intensity was consistently less than 5% of the mean intensity of the section (Figs. 3, 4, 5; Table 3). In addition, even though the level of expression varied from one cell type to another, we found that most cells within a given type had an equivalent and normally distributed level of clock protein expression (data not illustrated).

**Table 3:** Proportion of cells among identified retinal neurons that express the core circadian clock components at ZT02/06.

Cell type	Marker	Clock component					
		CLOCK	BMAL1	NPAS2	PER1	PER2	CRY2
Cones	*cArr*	++	+	+	+	+	+
		100±0%	100±0%	100±0%	96.33±3.66%	98.92±1.08%	100±0%
		(106/106)	(90/90)	(78/78)	(78/80)	(101/102)	(97/97)
Rods	*- cArr*	–	–	–	–	–	–
		0%	0%	0%	0%	0%	0%
		(0/2544)	(0/2410)	(0/2439)	(0/2426)	(0/3298)	(0/2213)
Horizontal cells	*CALD28*	++	++	++	+	+	+
		100±0%	100±0%	100±0%	98.25±1.75%	100±0%	81.25±3.61%
		(59/59)	(53/53)	(49/49)	(50/51)	(15/15)	(39/48)
Bipolar cells	*CHX10*	++	++	++	–	+	+
		100±0%	99.32±0.68%	99.12±0.88%	9.37±0.65%	99.15±0.85%	99.21±0.79%
		(136/136)	(137/138)	(111/112)	(11/117)	(117/118)	(121/122)
Bipolar cells	*PKCα*	++	++	++	–	+	+
		100±0%	100±0%	100±0%	7.04±0.69%	100±0%	100±0%
		(108/108)	(119/119)	(96/96)	(6/87)	(88/88)	(92/92)
Amacrine cells (all)	*PAX6*	++	++	++	+++	++	+
		100±0%	100±0%	100±0%	100±0%	100±0%	100±0%
		(76/76)	(71/71)	(64/64)	(64/64)	(73/73)	(69/69)
Dopaminergic AC	*TH*	+++	++	++	+++	+++	+++
		100±0%	100±0%	100±0%	100±0%	100±0%	100±0%
		(49/49)	(37/37)	(38/38)	(39/39)	(36/36)	(30/30)
Starburst AC (all)	*ChAT*	+	++	++	+	+	+
		100±0%	89.62±4.81%	100±0%	89.63±5.79%	81.50±6.67%	100±0%
		(52/52)	(46/51)	(58/58)	(48/54)	(46/57)	(49/49)
Starburst AC (off)	*ChAT*	+	+	++	+	+	+
		100±0%	81.02±3.24%	100±0%	78.52±0.74%	62.22±2.23%	100±0%
		(28/28)	(21/26)	(31/31)	(22/28)	(18/29)	(23/23)
Starburst AC (on)	*ChAT*	+	++	++	+	+	++
		100±0%	100±0%	100±0%	100±0%	100±0%	100±0%
		(24/24)	(25/25)	(27/27)	(26/26)	(28/28)	(26/26)
Ganglion cells	*Pax6*	++	++	++	++	++	++
		100±0%	100±0%	100±0%	100±0%	100±0%	100±0%
		(27/27)	(27/27)	(36/36)	(39/39)	(34/34)	(38/38)
Ganglion cells	*Brn3b*	++	++	++	++	++	++
		100±0%	100±0%	100±0%	100±0%	100±0%	100±0%
		(18/18)	(22/22)	(17/17)	(20/20)	(16/16)	(19/19)
ipRGCs	*Melanopsin (GFP)*	++	++	++	+++	++	++
		100±0%	100±0%	100±0%	100±0%	62.38±9.05%	100±0%
		(47/47)	(39/39)	(51/51)	(37/37)	(12/20)	(35/35)

Retinal sections were reacted with antibodies against a clock protein and a specific neuronal marker (see Table 1 for details about the antibodies). Clock protein expression within an identified retinal neuron was determined by measuring the mean fluorescence intensity within the boundaries of the cell soma labeled for a specific marker for all the somas within a section. Mean fluorescence in rods was determined within a large area (∼50×20 µm) selected in the ONL that did not include the cone somas. Significant clock protein expression was defined as fluorescent level higher than 150% background. Results are scored as: - (not significant, labeling intensity <150% background),+(150% background to 33% of maximum labeling intensity),++(33% to 66% of maximum labeling intensity),+++(66% to 100% of maximum labeling intensity). Note that almost all cells in the INL and GCL, which together with cones represent ∼20% of all retinal neurons, significantly expressed all of the clock proteins and that no significant expression was found for any clock protein in rods, which account for ∼80% of all retinal neurons. Data shown are averaged values from 3–5 animals (3 pictures from 3 different sections per animal) ± SEM.

**Figure 6:**
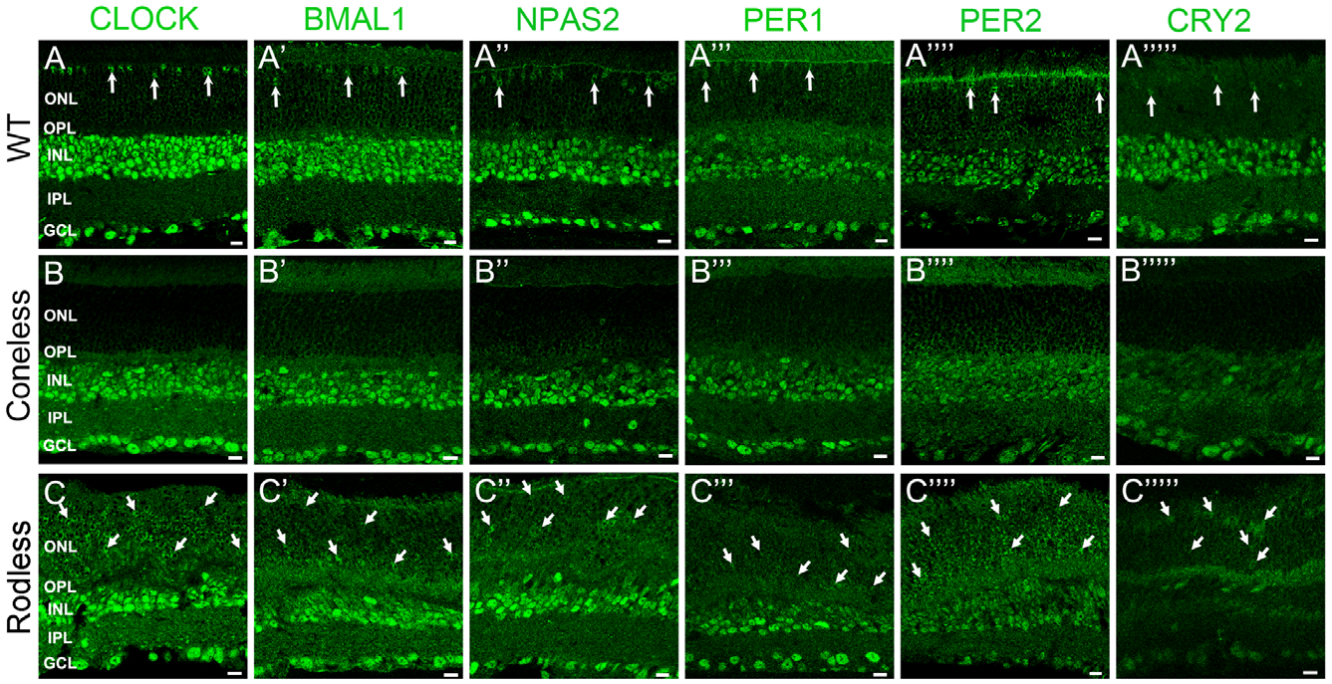
Core circadian clock protein expression in wild-type, coneless and rodeless retinas. Typical examples of vertical sections of wild-type C57Bl/6J (***A–A’””***), coneless (***B–B’””***), and rodeless (***C–C’””***) retinas immunolabeled for each of the following core clock proteins: CLOCK (***A–C***), BMAL1 (***A’–C’***), NPAS2 (***A”–C”***), PER1 (***A’”–C’”***), PER2 (***A””–C””***), and CRY2 (***A’””–C’””***). Retinal tissue was collected around ZT09. For a given clock protein antibody, confocal settings were adjusted on the brightest picture and the 2 other sections were taken at the same settings. Note that clock protein expression in the outer nuclear layer (ONL) is detected in a few cells in the wild-type retina (vertical arrows) and in most cells in the rodless retina (oblique arrows), but is very weak in the coneless retina. OPL: outer plexiform layer; INL: inner nuclear layer; IPL: inner plexiform layer; GCL: ganglion cell layer. Optical sections 3×0.4 µm. Bar is 10 µm.

Clock protein expression was further examined in retinas of mice that either lack cones (coneless) or rods (rodless) (Fig. 6). Both rodless and coneless retinas showed significant immunostaining in the inner retina comparable to the wild-type C57Bl/6J mouse (Fig. 1 and Fig. 6 top row). However, the level of immunofluorescence for any clock protein in the ONL of coneless retinas was very low (<5% of section mean fluorescence) (Fig. 6, middle row) whereas nearly all cells in the ONL of rodless retinas clearly expressed the clock components (Fig. 6, bottom row). The results are consistent with the observations in wild-type C57Bl/6J retinas and confirm that in the ONL, all six core circadian clock components are primarily expressed in cones and not in rods. We next analyzed whether undetectable levels of clock protein expression in rods at ZT02/06 represented the nadir of a daily rhythm or reflected a permanent low level of clock protein expression in rods.

### Daily and Circadian Rhythms of Expression of the Core Circadian Clock Proteins in Dopaminergic Amacrine Cells and Rod and Cone Photoreceptors

**Figure 7:**
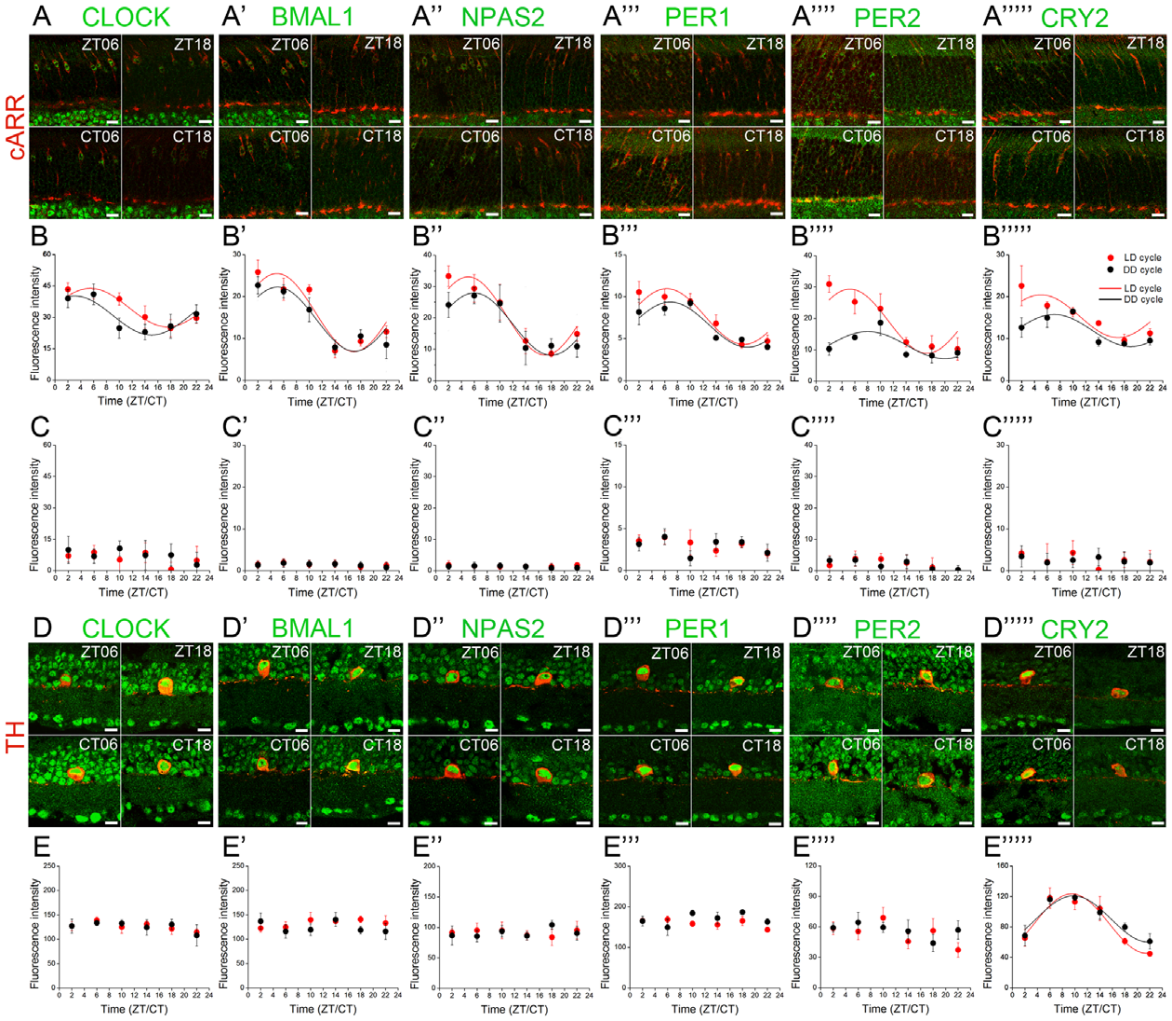
Circadian clock core component expression in mouse rod and cone photoreceptors and dopaminergic amacrine cells under LD and DD conditions. Typical examples of clock protein immunostaining in cones (***A*** *–* ***A*** *””’*) and dopaminergic cells (***D*** *–* ***D*** *’””*) obtained from retinas collected in the middle of the day (ZT06) or subjective day (CT06) or middle of the night (ZT18) or subjective night (CT18) are illustrated for CLOCK (***A*** *,* ***D***), BMAL1 (***A*** *’,* ***D*** *’*), NPAS2 (***A*** *”,* ***D*** *”*), PER1 (***A*** *’”,* ***D*** *’”*), PER2 (***A*** *””,* ***D*** *””*), and CRY2 (***A*** *’””,* ***D*** *’””*). COSINOR regression analysis (cosine curves) was performed only for clock protein levels displaying significant temporal variation (as determined by one-way ANOVA; *P*<0.05). The results from the COSINOR analysis are shown in Table 4. Note that the expression of all six clock proteins is rhythmic under LD and DD conditions in cones (***B*** *–* ***B*** *’””*) but arrhythmic under the same conditions in dopaminergic amacrine cells, except for CRY which is rhythmic under both LD and DD conditions (***E*** *–* ***E*** *’””*). Clock protein expression in rods remained low at any time point under both LD and DD conditions (***C*** *–* ***C*** *’””*). Each data point represents the mean fluorescence/identified neuron +/− SEM of 5/6 animals (5 pictures from 3–4 sections/animal).

To further define the spatial and temporal patterns of expression of the core clock proteins in the mouse retina, we analyzed the expression of each clock protein during a LD and a DD cycle in three specific cell types: the dopaminergic amacrine cells, rods, and cones. Each circadian clock protein was identified specifically in TH-positive amacrine cells in the inner retina or cARR-positive (cones) or -negative cells (rods) of the ONL. The intensity of immunolabeling of each clock protein in cones, rods, or dopaminergic cells was analyzed from 3–4 sections for each animal. The values from 5 to 6 animals of each clock protein at a given time were averaged. We found that at any given time point, the values of all labeled intensity were under normal distribution (or were distributed evenly), and, therefore, we are confident that the expression of these clock proteins displays a high degree of homogeneity among cells of the same type. The expression of all six clock proteins was rhythmic in cones under both LD and DD conditions, with the phase of the peak typically occurring around mid-day/subjective day (Fig. 7*A,B*; Table 4). The timing of the peak of expression of BMAL1 may appear at odds with the well-accepted model of the SCN clock mechanism, in which BMAL1 peak is anti-phase to the peak of expression of PER and CRY [2]. However, a similar observation has recently been reported in rat photoreceptors [37], [38], suggesting that the synchronous expression of the core clock genes and proteins may be a feature of the clock in mammalian photoreceptors (see Discussion for more detail). Surprisingly, the amplitude of the PER2 rhythm was ∼2-fold higher under LD compared to DD, indicating that daylight *per se* may increase PER2 expression in the cones (Fig. 7*A,B*; Table 4). Circadian clock protein expression in rods remained extremely low at any time point of the LD or DD cycle (Fig. 7*A,C*), suggesting that clock protein expression is constitutively low in these cells. Unexpectedly, only CRY2 expression was found rhythmic in the dopaminergic cells, whereas CLOCK, BMAL1, NPAS2, PER1 and PER2 expression remained constant and arrhythmic under either a LD or DD cycle (Fig. 7*D,E*; Table 4). Finally, no differences were observed in the dopaminergic cells between LD or DD conditions for any clock protein and in particular for PER2 (Fig. 7*D,E*; *Table 4*). These observations strengthen the view that the expression of the circadian clock components is constitutively low in rod photoreceptors. In addition, they demonstrate that the occurrence of both clock protein expression and of a rhythm of expression are neuronal cell-type dependent and that the coherence of clock protein expression within cells of the same type does not necessarily reflect the trend of the whole retina. These observations imply that analyses of the circadian organization and clock mechanism in the mammalian retina require cell-type specific investigations.

**Table 4:** COSINOR analysis of core circadian clock component expression in cones and dopaminergic amacrine cells.

Cell type/conditions	Clock component	a (mesor)	b (amplitude)	c (acrophase) (h)	*F*-value	*P*-value
Cones/LD						
	CLOCK	34.62±1.40	9.23±1.99	5.51±0.82	(*F*_3,27_) 209.90	<0.0001
	BMAL1	16.19±0.92	9.32±1.30	4.95±0.53	(*F*_3,27_) 121.44	<0.0001
	NPAS2	20.61±1.52	12.44±2.15	5.05±0.66	(*F*_3,27_) 72.74	<0.0001
	PER1	7.63±0.39	3.34±0.55	3.34±0.55	(*F*_3,27_) 138.76	<0.0001
	PER2	19.11±1.49	10.23±2.07	5.25±0.80	(*F*_3,23_) 62.32	<0.0001
	CRY2	15.34±0.99	5.12±1.40	5.04±1.04	(*F*_3,25_) 86.87	<0.0001
Cones/DD						
	CLOCK	30.86±1.87	9.36±2.64	3.01±1.08	(*F*_3,27_) 95.35	<0.0001
	BMAL1	14.60±1.01	7.66±1.42	5.06±0.71	(*F*_3,27_) 79.85	<0.0001
	NPAS2	18.02±1.64	9.91±2.32	5.99±0.90	(*F*_3,27_) 46.22	<0.0001
	PER1	6.66±0.33	2.70±0.46	6.88±0.66*	(*F*_3,21_) 148.89	<0.0001
	PER2	11.49±0.94*	4.28±1.30*	8.02±1.20*	(*F*_3,23_) 51.82	<0.0001
	CRY2	11.91±0.64*	3.84±0.89	7.03±0.92	(*F*_3,25_) 120.74	<0.0001
Dopaminergic cells/LD						
	CRY2	84.24±4.27	38.99±6.03	9.49±0.59	(*F*_3,27_) 143.87	<0.0001
Dopaminergic cells/DD						
	CRY2	90.57±3.48	30.35±4.93	9.79±0.62	(*F*_3,27_) 237.94	<0.0001

COSINOR regression analysis was performed on the data illustrated in Figure 7 and only for clock protein levels displaying significant temporal variation over the course of a LD or DD cycle (as determined by one-way ANOVA; *P*<0.05). The regression coefficients (a, b, and c) are given with their respective standard error estimates.

* : *P*<0.05 compared to respective LD value (Student *t*-test).

## Discussion

In this study, we have examined the expression of six key components of the mammalian circadian clock in mouse retina. Although our results globally agree with previous studies that reported widespread clock gene expression in retinal tissue, a general limitation of our study is that the experiments were performed by sampling *in vivo* retinas and therefore any rhythms or lack of could have been driven by signaling from other cells or tissues. Although *in vitro* experiments are more appropriate to demonstrate self-sustained rhythms, explanted retinal clocks may behave differently as a result of being kept outside of their normal environment. Our study is thus limited to the behavior of the *native* circadian clock proteins in mouse retinal neurons in *in vivo* conditions. In these conditions, we observed that: (1) among the photoreceptor cells, circadian clock core components are primarily expressed in cones and ipRGCs; (2) the occurrence of a rhythm of expression of clock proteins depends on the neuronal cell type, as illustrated by the differences between cones and dopaminergic cells; and (3) the expression of a given clock component appears to occur synchronously and to the same extent among retinal neurons of the same type. Thus, although our data support the view that retinal rhythms in the mouse retina originate from retinal pacemaker neurons, they also differ from the prevailing model in that they clearly indicate that the clockwork may operate differently in different retinal cell types.

### Which Photoreceptor Cell Types are Circadian Clocks?

Although clearly established in lower vertebrates [7], [8], the presence of a circadian clock in the *conventional* photoreceptors (i.e. rods and cones) of the mammalian retina has long been elusive. On the one hand, photoreceptor cells contain a circadian clock, because the rhythmic synthesis of melatonin takes place in the photoreceptor layer and persists *in vitro* [9], [10] even when photoreceptors are isolated from the inner retina [11]. On the other hand, the detection of rhythmic expression of clock genes in photoreceptors has been controversial; several studies have failed to detect this pattern [12], [13], [15], [17], [28], while with different techniques others have succeeded [11], [37], [38]. In addition, because cones represent only ∼3% of the photoreceptor cells in the mouse retina and their morphology is similar to rods [41], [42], it is not clear whether rods and/or cones express clock genes. Single cell PCR studies of six core clock genes in mouse rods reported no clock gene expression in 100% [17] and in 33% [12] of the cells tested. Out of the 66% of rods that expressed at least one clock gene, none (0%) expressed all six concurrently [12]. In the same study, a limited number of cells (eight) were identified as cone-like based on their morphology [12]. PCR results were similar to those obtained for rods. Together, results from these two studies converge towards the view that clock gene expression is very low in mouse rods. The weak level of clock protein expression we found in rods agrees with these studies but prevented us from determining whether clock genes were differentially expressed in subpopulations of rods, as suggested by the Ruan et al. study [12]. However, considering the limitations of single-cell molecular biology, the small sample size and the lack of information about the collected cells, it is hard to make a strong case for or against the presence of a clock mechanism in cones based on the Ruan et al. study. Here, we found strong rhythmic expression of six core components of the mammalian circadian clock in cone photoreceptors (Fig. 7). In contrast, we found only limited expression, if any, in rods (Fig. 7). Although we cannot rule out that core clock proteins are expressed in rods below detectable level, our observations agree with previous studies that reported limited clock gene expression in the photoreceptor layer [11], [37], [38] or in isolated rod photoreceptors [12], [17]. Together with these studies, our data suggest that most of the clock gene expression found in the photoreceptor layer with sensitive techniques that required nucleic acid amplification may have originated from cones and not from rods and that cones are likely autonomous circadian clock cells that drive rhythms in the photoreceptor layer. The control of circadian rhythms in rods by the cone circadian clock could involve the nighttime-restricted diffusion of substances through the rod-cone gap junction [43] and/or paracrine loops involving melatonin and/or dopamine [5], [44] or other means. The presence of a clock mechanism in cones and not rods is consistent with the observation that light-entrainment of retinal rhythms in vertebrates requires bright (photopic) light [7], [8], [9], [13], [45], [46].

Although the clock in cones resembles that in the SCN in rhythmically expressing circadian clock proteins [2], [22], it differs from the SCN clock in several aspects. In particular, CLOCK expression is rhythmic in cones while it is constitutively arrhythmic in the SCN [2]. More surprisingly, expression of all six core clock proteins is coordinated in cones with a peak during the daytime (Fig. 7) whereas in the SCN, the peak in PER and CRY proteins occurs late in the day/early night (∼ZT/CT 12), and the BMAL1 peak is antiphase and occurs in the late night/early morning (∼ZT/CT 0) [2]. Two recent studies in the rat retina have shown that *Clock/Bmal1* transcripts peak in phase with *Per/Cry* transcripts in photoreceptors [37], [38].

The reported concomitant increase in clock gene expression at night in the photoreceptor layer is consistent with the increase in clock protein expression in cones during the daytime that we report here. The circadian expression of *Bmal1* is influenced by nuclear receptors of the ROR family and Rev-ERBα, which target a ROR-response element in the promoter of the *Bmal1* gene [2]. RORs and REV-ERBα compete for binding the *Bmal1* promoter, with RORs activating while REV-ERBα repressing *Bmal1* expression. It has been shown that tissue-specific variation of the ratio of expression between these two families of trans-regulatory elements impinges on the rhythmic expression of *Bmal1* in a tissue-dependent manner [2]. ROR family members also control *Npas2* (and to some extent *Clock*) expression [2]. Interestingly, Sandru et al. [38] reported a strong rhythm of *Rorβ* in rat photoreceptors with an increased expression at night and a much smaller-amplitude rhythm in *Rev-ERBα* under a LD cycle that did not persist under DD conditions. It is tempting to speculate that the nighttime peak in *Rorβ* might shift the balance of modulators of *Bmal1* expression in favor of ROR activators and result in an increase in *Bmal1*, *Clock* and *Npas2* expression towards the end of the night [38]. This would translate into a peak in proteins 4–6 hrs later- that is around midday. This hypothetical model would require in particular that BMAL1 and CLOCK or NPAS2 were not rate-limiting in the transcriptional activation of the *Per* and *Cry* genes. In addition, other factors, such as cAMP, whose intracellular levels increase at night, may play a role as well in framing the temporal expression of the clock genes in cones [5]. Although many details remain to be clarified, our study together with others [37], [38] clearly indicates that a unique circadian clockwork is expressed in mammalian cones.

We also report that all six core elements of the mammalian clock are expressed in ipRGCs (Fig. 5; Table 3). This type of ganglion cell expresses the photopigment melanopsin and is considered a third class of photoreceptor in mammals [39], [40]. Although a previous study did not find detectable expression of *Per1* in ipRGCs [15] the expression of clock components in ipRGCs is consistent with a body of indirect evidence that links ipRGCs with several circadian rhythms within the eyes and the SCN. These include (1) a circadian rhythm in melanopsin expression [47]; (2) a biochemical circadian rhythm in the SCN-onto which the ipRGC project- that disappears following enucleation [48]; (3) the circadian modulation of ipRGC intrinsic light responses [49], [50]; and (4) a role for ipRGCs in the circadian modulation of the ERG [51]. Although these studies, together with our discovery of clock component expression in ipRGCs (Fig. 5; Table 3) strongly suggest that ipRGCs may be autonomous circadian clocks, the possibility that some other oscillators in the retina may drive rhythms in ipRGCs has been proposed as well [52], [53]. Thus even if our observations and others suggest that ipRGCs are circadian clocks, we must be cautious in interpreting these results. The functional role of the core clock components in ipRGCs remains to be firmly established.

### Are Dopaminergic Amacrine Cells Autonomous Circadian Clocks?

Numerous studies have consistently reported the existence of clock gene and protein expression in inner retinal cells [12], [15], [16], [17], [28]. Clock gene expression in the inner retina persists *in vitro* even in the absence of rod and cone photoreceptors, supporting the view that some cells in the inner retina are circadian clocks that function autonomously and independently of the photoreceptor clock [12], [13], [14]. Even though most cells of the inner retina express the core clock components (Figs. 1, 2; Table 2), we found that five out of six clock proteins were constitutively expressed in the dopaminergic amacrine cells (Fig. 7*E*). In addition, no difference in clock protein expression level was observed between LD and DD conditions in dopaminergic cells (Fig. 7*E*), while PER2 expression in the cones (Fig. 7*B*) was clearly higher during the light phase of the LD cycle compared to the subjective day of the DD cycle (Table 4). Light-entrainment is a key property of circadian clocks, which relies on the induction of clock gene and protein expression. Light-induced expression in *Per* genes and/or proteins has been reported in vertebrate retinas [30], [31], [45], [46], [54]. Together with the arrhythmic expression of most clock proteins, the absence of light-sensitivity of clock protein expression in the dopaminergic cells would argue against the presence of a functional circadian clock in these cells. In addition, to date no circadian phenotype has been linked to the clock mechanism within the dopaminergic cells, as the circadian rhythm in dopamine release requires the presence of a melatonin rhythm [5]. Thus, while confirming the expression of circadian clock components in dopaminergic cells, our data expose differences in the clock mechanism between cones and dopaminergic cells and question the cell autonomy of the later.

However, dopaminergic amacrine cells could play a key role in the temporal organization of retinal function through the release of dopamine. Dopamine release is under the dual control of a circadian clock and light [55], with a peak during the day under light-adapted conditions and a trough at night in the dark, and is known to synchronize retinal rhythms [7], [8], [45], [46], including in mammals [13]. Thus, whether retinal dopamine neurons are autonomous circadian clocks remains an open question, but these cells may play a greater role in participating in the light entrainment of retinal rhythms and synchronization among retinal neurons rather than in initiating retinal pacemaker activity. In a way, retinal dopaminergic neurons may play a role similar to the retinocepient vasointestinal neuropeptidergic neurons of the SCN [2], [18].

### A General Organization of Mammalian Circadian Cocks?

Our observations in the retina may represent the case of more general organization of circadian clock cells that also occur in other tissues. For instance, emerging views for the organization of SCN rhythmic function emphasizes the strong heterogeneity in the individual properties of its cell-autonomous circadian oscillators and the important role of neuronal networks in shaping a robust and coherent rhythmic output [18]. However, although most SCN neurons express the core clock components, a link between specific SCN neuronal subtypes and rhythmic properties has been difficult to establish, and the means through which networks synchronize cellular oscillators and rhythmic outputs are still unclear [18]. By demonstrating that clock components are widely expressed among retinal neurons and that a high degree of heterogeneity in their expression occurs among retinal cell types, our results suggest that the organization of populations of clock cells in retinal tissue may share similar features with that of the SCN. Dissecting the clock mechanism on a cell-type basis in the retina will thus likely shed light not only on the circadian organization of retinal function but also on the general organization of circadian clocks in mammalian tissues.
